# COVID‐19 infection and Leser–Trelat sign: Is there an association?

**DOI:** 10.1002/ccr3.7638

**Published:** 2023-07-04

**Authors:** Farhad Handjani, Roya Radanfar, Mozhdeh Sepaskhah, Niloofar Dehdari Ebrahimi

**Affiliations:** ^1^ Molecular Dermatology Research Center, Shiraz University of Medical Sciences Shiraz Iran; ^2^ Department of Dermatology Shiraz University of Medical Sciences Shiraz Iran; ^3^ Student Research Committee Shiraz University of Medical Sciences Shiraz Iran

**Keywords:** COVID‐19, infection, keratosis, Leser–Trelat sign, seborrheic

## Abstract

**Key Clinical Message:**

The etiology of the Leser–Trélat sign is still unknown, it is likely that viral infections like COVID‐19 can be associated with eruptive seborrheic keratosis, although the exact pathogenesis is still not clear, but this phenomenon can be due to TNF‐alpha and TGF‐alpha and immunosuppression condition as well as in COVID‐19 infection.

**Abstract:**

Seborrheic keratosis is a typical benign skin lesion that is almost always seen in elderly populations. The sudden increase in size or an increase in the number of these lesions is called Leser–Trelat sign, this sign is suggesting as a paraneoplastic appearance of internal malignancy. But, Leser–Trelat sign is also described in some nonmalignant conditions, for example, human immunodeficiency virus infection and human papillomavirus infection. Herein, we describe a patient with Leser–Trelat sign after recovery from COVID‐19 infection with no evidence of internal malignancy. This case was partially presented as a poster in the 102nd Annual Congress of British Association of Dermatologists in Glasgow, Scotland from July 5 2022 to July 7 2022. British Journal of Dermatology, 187, 2022 and 35. The patient signed written informed consent to permit the publication of the case report without identifying data and to use the photography for publication. The researchers committed to maintaining patient confidentiality. Institutional ethics committee approved the case report (ethics code: IR.sums.med.rec.1400.384).

## Graphical abstract

Dermoscopic examination of eruptive papules after COVID‐19 infection revealed the brown lesion with milia‐like cysts, brown dots, and multiple comedone‐like openings.

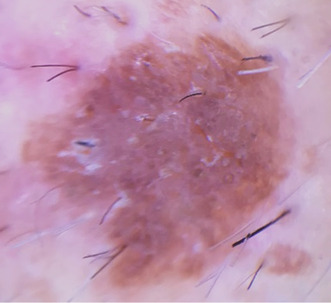

## 1 INTRODUCTION

COVID‐19 infection is caused by severe acute respiratory syndrome coronavirus 2 [SARS‐CoV‐2], a betacoronavirus. The most common clinical manifestations of COVID‐19 are respiratory symptoms. As the pandemic progressed, other aspects were discovered, including cutaneous manifestations of the disease^1^ for example erythematous rash, urticaria, vesicle formation, and purpura.^2^

To date, no report has been made of the sudden eruption of seborrheic keratoses following COVID‐19 infection.

The sudden increase in size or an increase in the number of these lesions is called Leser–Trelat sign, this sign is suggesting as a paraneoplastic appearance of internal malignancy.^3^ The most frequently associated malignancies are adenocarcinoma of the colon, stomach, lung, or breast, although the Leser–Trélat sign has also been reported in nonmalignant conditions, for example, lepromatous leprosy,^4^ erythrodermic pityriasis rubra pilaris,^5^ human immunodeficiency virus infection,^6^ and human papillomavirus infection.^7^ Leser–Trélat sign can also occur in healthy individuals in the absence of internal malignancy.^8^ Herewith, we describe a case of Leser–Trelat sign, which presented after recovery from COVID‐19 infection in a 50‐year‐old man.

## 2 CASE REPORT

A 50‐year‐old man was referred to our dermatology clinic complaining of sudden appearance of multiple warty‐like lesions on his back which had occurred 2 months after recovery from COVID‐19 infection.

According to his medical history, the patient presented with dyspnea, fever, and cough, about 2 months prior to the appearance of the skin lesions. He was referred to a health center where a nasopharyngeal swab was taken, and his PCR test for COVID‐19 was positive. In addition, bilateral patchy ground‐glass infiltration was reported in his high‐resolution computed tomography (HRCT) scan in favor of COVID‐19 infection. The patient was then treated with acetaminophen, dexamethasone (intramuscular injection), salmeterol, and fluticasone inhaler, and his symptoms improved.

**FIGURE 1:**
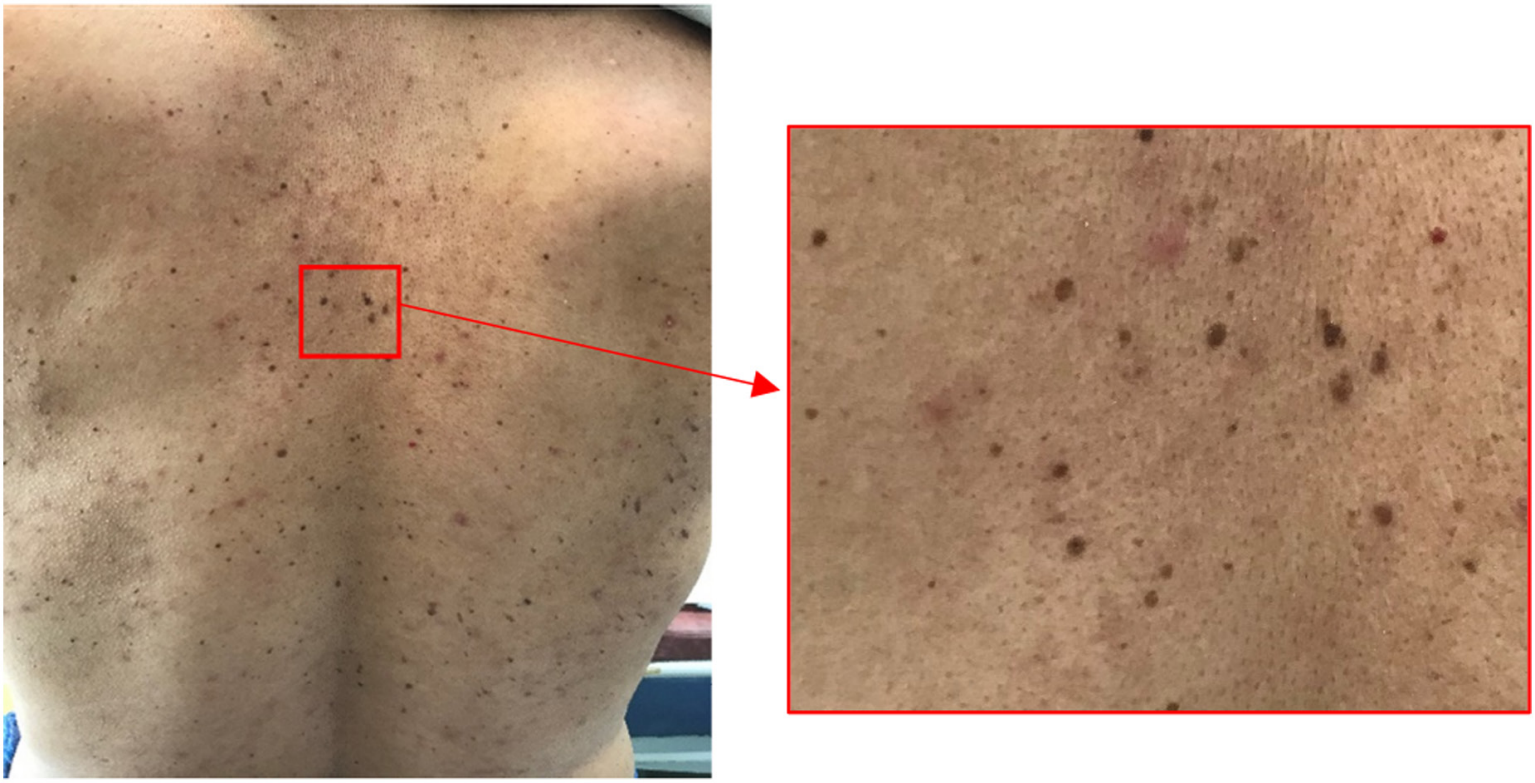
Small pigmented verrucous papules on the patient's back.

**FIGURE 2:**
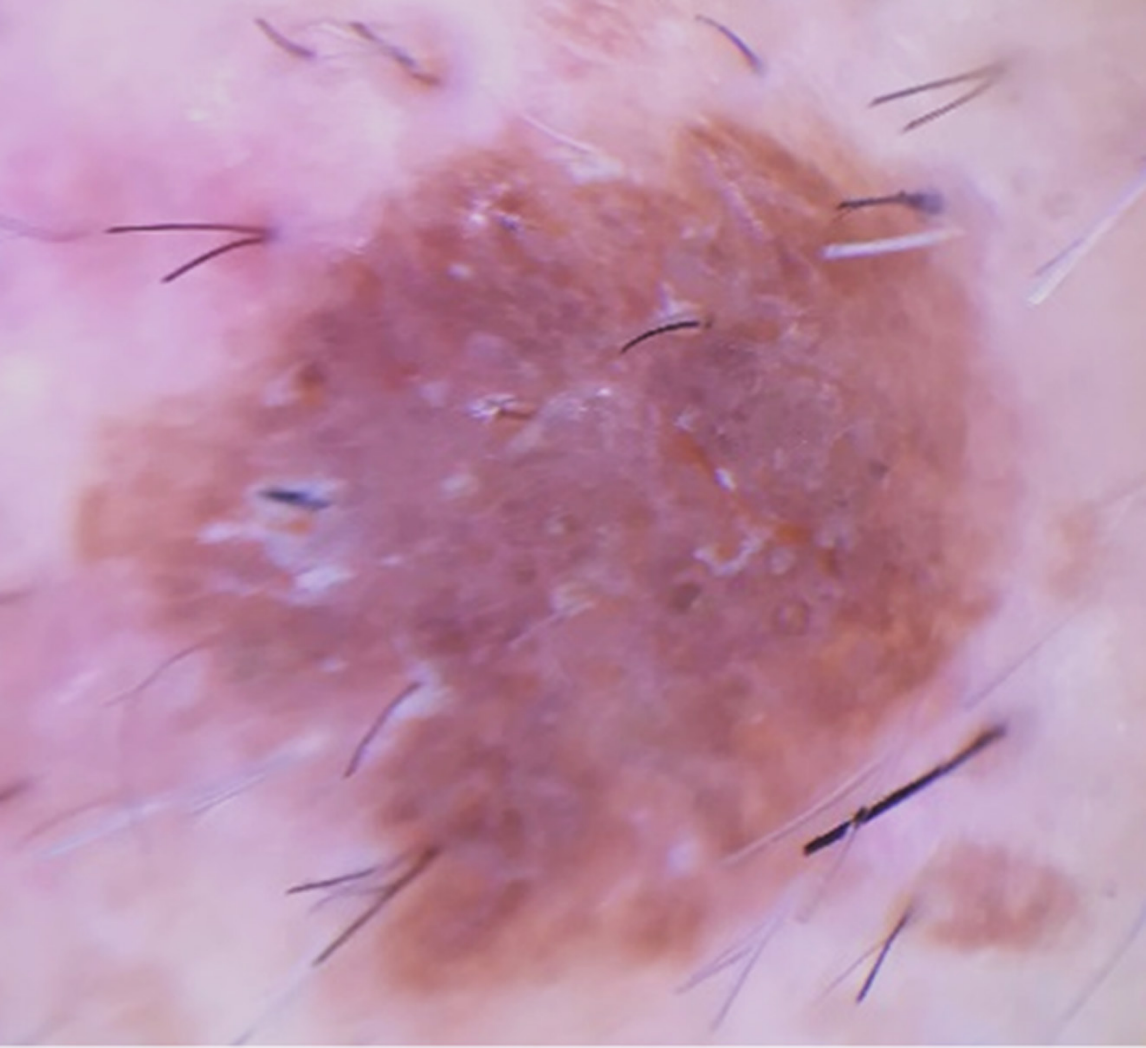
Dermoscopic examination revealed a brown lesion with milia‐like cysts, brown dots, and multiple comedone‐like openings.

**FIGURE 3:**
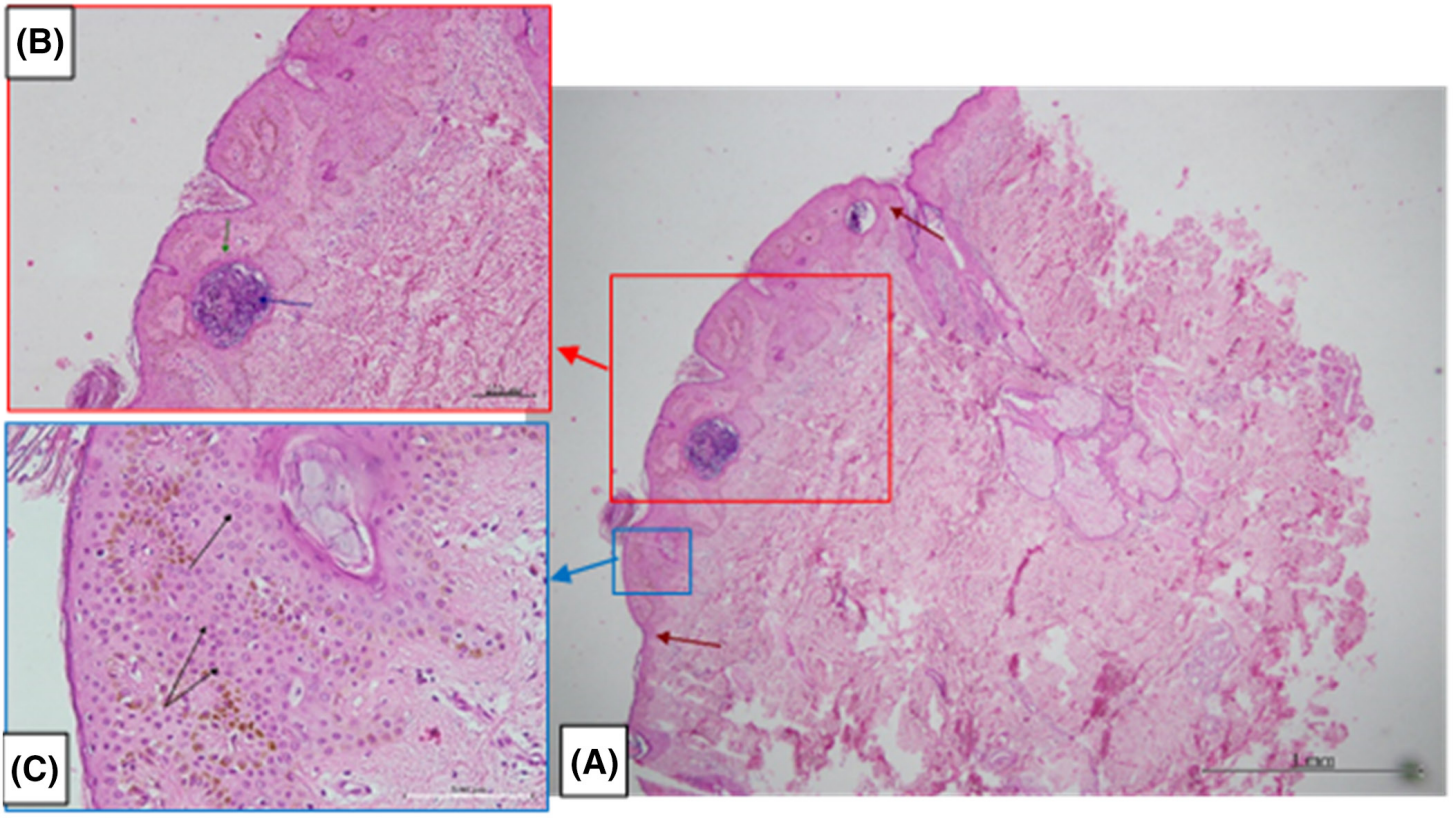
Histopathology examination: (A) Hyperkeratosis, well‐defined epidermal hyperplasia (defined between red arrows), and horn cysts (hematoxylin and eosin stain, 40 ×); (B) Hyperkeratosis, epidermal hyperplasia, horn cyst (blue arrow), surrounded by basaloid cells (green arrow), and basal melanosis (hematoxylin and eosin stain, 100 ×); (C) Epidermal acanthosis, predominantly composed of basaloid cells (black arrows) and some squamoid cells. Increased melanin is seen mostly in the dermo–epidermal junction (hematoxylin and eosin stain, 400 ×).

Two months after recovery from mild COVID‐19 infection, multiple small asymptomatic pigmented warty‐like papules appeared on the patient's back. Physical examination revealed multiple rough, oval‐shaped, brownish papules varying in size from 2 mm in diameter to 15 × 5 × 2 mm (Figure 1). Dermatoscopy of the lesions was also performed. Both clinical and dermoscopic findings were in favor of seborrheic keratosis (Figure 2). In order to reach a final diagnosis, a skin biopsy was requested, and microscopic examination of the biopsy specimen showed hyperkeratosis, well‐defined epidermal hyperplasia, composed mainly of the proliferation of benign‐looking basaloid cells and fewer squamoid cells, horn cysts, and increased melanin, mostly in the dermo–epidermal junction. The dermis showed no significant change (Figure 3). Based on the above findings, the patient was diagnosed with eruptive seborrheic keratosis.

To determine the possible cause of this eruption, the patient was further evaluated. In his past medical history, he was generally healthy before his COVID‐19 infection and had no history of comorbidities. The patient was then examined to rule out any internal malignancies. Laboratory tests revealed normal results and included a complete blood count (white blood cells 5300/mm^3^, red blood cells: 4.5 × 10^6^/mm^3^, platelets: 152000/mm^3^), liver and kidney function tests, electrolytes, prostate‐specific antigen, and urine analysis. Gastrointestinal endoscopy and colonoscopy ruled out any gastrointestinal malignancy. Chest X‐ray and high‐resolution computed tomography (HRCT) scan revealed no malignant lesion. In addition, the patient's abdominopelvic sonography was normal. The patient had no family history of similar skin lesions and gave no history of any chronic inflammatory skin diseases or viral conditions. Therefore, the appearance of the Leser–Trelat sign after COVID‐19 infection could be regarded as a possibility in this patient.

## 3 DISCUSSION

Seborrheic keratosis is a typical benign skin lesion that is almost always seen in elderly populations, the sudden increase in size or an increase in the number of these lesions is called Leser–Trelat sign. The etiology of the Leser–Trélat sign is still unknown, although it has been considered as a paraneoplastic phenomenon. The most frequently associated malignancies are adenocarcinoma of the colon, stomach, lung, or breast. Some cases with the Leser–Trélat sign have occurred in nonmalignant patients, including patients with underlying infections like viral infections,^6^, ^7^, ^15^ lepromatous leprosy,^4^ in association with erythrodermic pityriasis rubra pilaris.^5^ In addition, a case has been reported in a heart transplant patient treated with immunosuppressive drugs.^9^

In addition, some observations have indicated that the Leser–Trélat sign may develop following viral infections. Inamadar and Palit^6^ have reported a case with human immunodeficiency virus infection who developed the Leser–Trélat sign. In a study by Tsambaos et al., human papillomavirus (HPV) DNA was positive in 34 patients from among 173 cases with nongenital seborrheic keratosis.^7^ Gushi et al. evaluated 104 nongenital seborrheic keratosis immunopotent patients for HPV DNA and showed that 30 of 104 seborrheic keratosis samples contained HPV DNA.^15^

Some theories have associated its development to transforming growth factor (TGF)‐alpha and epidermal growth factors secreted from tumor cells. According to previous studies, TGF‐alpha is overexpressed in seborrheic keratosis, and it may play a significant role in the progression and increase in the number of seborrheic keratoses.^10^, ^11^ It has been shown that COVID‐19 infection with lung injury can induce expression of TGF,^12^ so there is the possibility of a similar mechanism in our patient, although severe lung findings were not reported. However Leser–Trelat sign is usually associated with a variety of immune suppression conditions such as malignancy or viral infection, so development of eruptive seborrheic keratosis may be due to immunosuppression situation caused by COVID‐19 infection and not exactly due to TGF‐alpha.

Immunohistochemical analysis has also revealed an increased expression of tumor necrosis factor‐alpha (TNF‐alpha) in seborrheic keratosis skin lesions,^13^ which is in accordance with increased inflammatory cytokines such as TNF‐alpha observed in COVID‐19 patients.^14^

Although, the development of Leser–Trelat sign in healthy persons does not fully support the theories of TNF‐alpha and TGF‐alpha and immunosuppression conditions.

Therefore, it is likely that viral infections like COVID‐19 can be associated with eruptive seborrheic keratosis, although the exact pathogenesis is still not clear.

## AUTHOR CONTRIBUTIONS

**Farhad Handjani:** Conceptualization; investigation; supervision. **Roya Radanfar:** Conceptualization; data curation; writing – original draft. **Mozhdeh Sepaskhah:** Data curation; investigation; supervision; writing – original draft. **Niloofar Dehdari Ebrahimi:** Conceptualization; data curation; writing – original draft.

## FUNDING INFORMATION

There is no funding source for this scientific work.

## CONFLICT OF INTEREST STATEMENT

The authors have no conflicts of interest to declare. All co‐authors have seen and agree with the contents of the manuscript and there is no financial interest to report.

## Data Availability

The data that support the findings of this study are available on request from the corresponding author. The data are not publicly available due to privacy restrictions.

## References

[1] P11: Leser‐Trélat sign: can it follow COVID‐19 infection? British Journal of Dermatology. 2022;187(S1):35‐36. doi:10.1111/bjd.21133

[2] Recalcati S . Cutaneous manifestations in COVID‐19: a first perspective. J Eur Acad Dermatol Venereol. 2020;34(5):e212‐e213. doi:10.1111/jdv.16387 PubMed PMID: 32215952. 32215952

[3] Bernett CN , Schmieder GJ . Leser Trelat sign. StatPearls. Treasure Island (FL): StatPearls Publishing Copyright © 2021, StatPearls Publishing LLC; 2021. 29261959

[4] D'Souza M , Garg BR , Reddy BS , Ratnakar C . Lepromatous leprosy with extensive truncal seborrheic keratoses and acral verruca vulgaris. Int J Dermatol. 1994;33(7):498‐500. doi:10.1111/j.1365-4362.1994.tb02864.x PubMed PMID: 7928035. 7928035

[5] Schwengle LE , Rampen FH . Eruptive seborrheic keratoses associated with erythrodermic pityriasis rubra pilaris. Possible role of retinoid therapy. Acta Derm Venereol. 1988;68(5):443‐445. PubMed PMID: 2461032. 2461032

[6] Inamadar AC , Palit A . Eruptive seborrhoeic keratosis in human immunodeficiency virus infection: a coincidence or 'the sign of Leser‐Trélat'? Br J Dermatol. 2003;149(2):435‐436. doi:10.1046/j.1365-2133.2003.05463.x PubMed PMID: 12932267. 12932267

[7] Tsambaos D , Monastirli A , Kapranos N , et al. Detection of human papillomavirus DNA in non‐genital seborrhoeic keratoses. Arch Dermatol Res. 1995;287(6):612‐615. doi:10.1007/bf00374085 PubMed PMID: 7487151. 7487151

[8] Safa G , Darrieux L . Leser‐Trélat sign without internal malignancy. Case Rep Oncol. 2011;4(1):175‐177. doi:10.1159/000327363 PubMed PMID: 21526136; PubMed Central PMCID: PMCPMC3081649 Epub 20110329. 21526136 PMC3081649

[9] Hsu C , Abraham S , Campanelli A , Saurat JH , Piguet V . Sign of Leser‐Trélat in a heart transplant recipient. Br J Dermatol. 2005;153(4):861‐862. doi:10.1111/j.1365-2133.2005.06850.x PubMed PMID: 16181485. 16181485

[10] Ellis DL , Kafka SP , Chow JC , et al. Melanoma, growth factors, acanthosis nigricans, the sign of Leser‐Trélat, and multiple acrochordons. A possible role for alpha‐transforming growth factor in cutaneous paraneoplastic syndromes. N Engl J Med. 1987;317(25):1582‐1587. doi:10.1056/nejm198712173172506 PubMed PMID: 2825016. 2825016

[11] Xiao H , Sheng W , Song J . Expression of TGF‐α and EGFr in seborrheic keratosis. Medical Journal of Wuhan University. 2004;25(3):309‐310.

[12] Ferreira‐Gomes M , Kruglov A , Durek P , et al. SARS‐CoV‐2 in severe COVID‐ induces a TGF‐β‐dominated chronic immune response that does not target itself. Nat Commun. 2021;12(1):1961. doi:10.1038/s41467-021-22210-3 PubMed PMID: 33785765; PubMed Central PMCID: PMCPMC8010106 Epub 20210330. 33785765 PMC8010106

[13] Chen XY , Yan BX , Man XY . TNFα inhibitor may be effective for severe COVID‐19: learning from toxic epidermal necrolysis. Ther Adv Respir Dis. 2020;14:1753466620926800. doi:10.1177/1753466620926800 PubMed PMID: 32436460; PubMed Central PMCID: PMCPMC7243041. 32436460 PMC7243041

[14] Manaka L , Kadono S , Kawashima M , Kobayashi T , Imokawa G . The mechanism of hyperpigmentation in seborrhoeic keratosis involves the high expression of endothelin‐converting enzyme‐1alpha and TNF‐alpha, which stimulate secretion of endothelin 1. Br J Dermatol. 2001;145(6):895‐903. doi:10.1046/j.1365-2133.2001.04521.x PubMed PMID: 11899142. 11899142

[15] Gushi A , Kanekura T , Kanzaki T , Eizuru Y . Detection and sequences of human papillomavirus DNA in non‐genital seborrhoeic keratosis of immunopotent individuals. J Dermatol Sci. 2003;31(2):143‐149. doi:10.1016/s0923-1811(03)00002-1 PubMed PMID: 12670725. 12670725

